# A Case Relating to the Influence of Maternal Impressions upon the Fœtus

**Published:** 1891-05

**Authors:** R. Osgood Mason

**Affiliations:** New York.


ARTICLE III.

### A CASE RELATING TO THE INFUENCE OF MATERNAL IMPRESSIONS UPON THE FŒTUS.

BY R. OSGOOD MASON, M. D., NEW YORK.

The *Record* for May 18, 1889, contained a communication from me intended to show that physicians and others, who believe that strong impressions made upon the mother during pregnancy may have an influence upon the fœtus *in utero*, even to the extent of causing marked physical changes, need not necessarily be believers in *witch broth* and such like supernatural things, but may be well-informed, thoughtful, and even scientific people. While preparing that communication a newspaper item fell under my observation referring to a family of albino children in Harrisburg, Pa., presenting some interesting features bearing upon the subject in hand, and having the authority of the attending physician, Dr. M. K. Bowers, of the same city. I at once wrote to the doctor in regard to the matter; he kindly replied, and I found in him a most interested and intelligent observer and correspondent.

The following facts were elicited which, with the doctor's permission, I have prepared for publication.

Two years ago, when our correspondence commenced, the family referred to consisted of father, mother and three albino children. The father of the children is a very dark-skinned man with black eyes and jet-black hair; the mother is not quite so dark, but has dark hair and dark blue eyes. The grandparents all have dark complexions and dark hair, and the family, as far back as its history can be traced, is entirely free from freaks or abnormalities of any kind.

Two years previous to the birth of the first albino the mother gave birth to a perfectly normal, healthy child having dark eyes and very black hair. This child died just previous to her next pregnancy.

With the second pregnancy everything went on as usual until near the beginning of the second month, when one day she unexpectedly received a small box by express from a friend in Philadelphia. She was quite ignorant of the contents of the box, which, however, was at once opened in her presence and was found to contain a large, handsome, perfect albino rat. As soon as she saw it she became nervous and declared that the sudden impression made upon her would show itself upon her child.

The rat, however, was tame, and was at once domiciled in the house. It became a general pet in the family, the pregnant woman herself also becoming very fond of it, often playing with it and holding it in her hands. A short time before her confinement the rat was accidentally killed, an incident which caused her considerable annoyance and sorrow.

A few days later the doctor was called to the confinement, which resulted in the birth of a fine boy baby, but a perfect albino. The mother, when informed of the fact, did not seem at all surprised, but said at once, "I knew it, I told you so. It was the rat—oh, that rat!" However, the boy grew beautifully and became a great pet both at home and abroad, "the mother herself becoming *wonderfully* attached to him."

The mother soon became pregnant again, and informing the doctor of the fact, she remarked that she had made up her mind to have a pair of albinos. Her expectations were realized, and in due time she was delivered of a fine girl baby, also a perfect albino.

Upon the occurrence of her next pregnancy, she remarked that this ought to be a black-haired baby, but she feared she would be disappointed. Her fears proved well-founded, and her pregnancy resulted in another perfect albino girl.

So, dating from the white rat episode, we have three perfect albino children, concerning whom the doctor writes: "The children are remarkable for their health, never having been sick, general activity, and unusual intelligence. They are much better specimens of the albino type than any found in museums or circus shows. They have snow-white hair, eyebrows, and lashes, the hair being remarkably fine and soft. The eyes are extremely sensitive to light, and when exposed to the sunlight or a bright artificial light they keep them closed; but in a subdued light or on a cloudy day, they are wide-awake and have very acute vision, being able to see the smallest objects, even when the room is quite dark. The sclerotic is of a dull white, a distinct bright pink (almost red) line binding its juncture with the cornea. The pupil is of a slightly darker shade of pink. The eyes are constantly in motion, never resting more than a moment upon any one object. The two older children are extremely bright and active, and the new-comer, now four days old, promises to rival her distinguished brother and sister. She is an unusually fine baby."

Such was the state of affairs in the albino family in May, two years ago.

During the second week of April last I received another letter from Dr. Bowers, from which I gather the following interesting facts. The albino children were all living and in excellent health; furthermore, on Sunday morning, April 5th, the doctor was again called to attend the mother in her confinement, when she gave birth to a perfect and vigorous twelve-pound boy, with black hair and dark-blue eyes, as utterly unlike the albinos as it was possible to imagine. The three former children are, if possible, more pronounced albinos than they were two years ago, and the family, as at present constituted, presents a most singular and interesting group.

The doctor then goes on to say: "This last dark-haired, normal child appears to be a striking example of will-power exerted by the mother. She has been under my care continually, and I know that it has been her one chief desire to have such a child. She was exceedingly anxious to become pregnant, and when she was convinced that such was the case she sent for me, and during our conversation made this remark: 'Now, doctor, keep your eye on me, for I am sure it is all right, and this time it will be a black-head!' She at once sent her albino children away to her mother's house, to remain during her pregnancy, and her husband tells me that her one constant song and desire was for the dark-haired baby. She talked of it constantly, she even talked of it in her sleep." When called to attend her she at once said to the doctor, "Now I am ready for my dark baby."

The doctor remarked that the desire on the part of his patient for a dark-haired child was not merely the whim of an ignorant or illiterate person; on the contrary, she was well educated, generally intelligent, and an unusually brilliant woman. Her parents were educated people—her father at present holding a prominent position in one of the offices of the State Government—and that her desire was accompanied by a very strong faith and intelligent effort for its fulfilment.

The case is simply one, and a strong one, bearing on the question of the possible effect of maternal impressions upon the fœtus *in utero*.

The case sums up as follows: Parents both dark—one *very* dark, to whom is born, as might naturally be expected, a dark-complexioned child.

Early in the second pregnancy of the mother she is surprised and made nervous by the sudden and unexpected appearance in her family of a large, handsome albino rat. She affirms that it will have an effect upon her unborn child.

Albino number one is born. She becomes exceedingly fond of it; its peculiar face is ever before her. She is not averse to another similar child, but desires it.

Albino number two is born. The next pregnancy she hopes may terminate by the birth of a dark-haired child; but she is surrounded by the same influences, and the same models are before her. She fears disappointment, and she experiences it.

A third albino child is born. Upon the occurrence of her next pregnancy she is thoroughly aroused, and is influenced by a peculiarly strong wish for a dark haired child. She affirms that it will be so. She exerts her will to promote her wish. She removes from her sight the models which she does not wish should influence her. She directs her mind constantly to the ideal of a dark-haired child. She dreams of her dark-haired child and speaks of it in her sleep—an important fact as indicating unusual susceptibility to impressions. She feels that she will not be disappointed, and she is not. A perfect dark-haired child is born.

The "coincidence" man is abroad, but he has a sallow and antiquated look, and along with his friend, the "unconscious muscular action" man, seems to belong more properly to the early part of the century. Experimental psychology is bringing to light many important facts showing mind producing physical effects beyond the limit of actual nerve-supply. Why not in cases like that above cited? I think we have occasion to thank Dr. Bowers for his interesting case in point.—*Medical Record.*

